# Editorial: Wheeled Mobility Biomechanics

**DOI:** 10.3389/fbioe.2016.00053

**Published:** 2016-06-28

**Authors:** Philip Santos Requejo, Jill L. McNitt-Gray

**Affiliations:** ^1^Rehabilitation Engineering, Rancho Los Amigos National Rehabilitation Center, Downey, CA, USA; ^2^Biomechanics Research Laboratory, Department of Biological Sciences, University of Southern California, Los Angeles, CA, USA; ^3^Biomechanics Research Laboratory, Department of Biomedical Engineering, University of Southern California, Los Angeles, CA, USA

**Keywords:** wheelchair, mobility, shoulder, pain, spinal cord injuries, propulsion, car transfer, ultrasonography

**The Editorial on the Research Topic**

**Wheeled Mobility Biomechanics**

For the manual wheelchair (MWC) user, loss of lower extremity function often places the burden for mobility and activities of daily living on the upper extremities. People who use a MWC commonly report fatigue and musculoskeletal pain in the shoulder, most often due to increased demands of mobility (Kemp and Mosqueda, 2004; Kemp, 2005). Because individuals who rely on MWC are dependent on their upper extremities for mobility and requisite activities (sitting, transfers, and pressure reliefs), as well as activities initiated from the wheelchair (exercise, reaching, and lifting), shoulder pain and dysfunction (Vissers et al., 2008; Mulroy et al., 2011a) can limit independence and functional mobility (Gerhart et al., 1993; Pentland and Twomey, 1994; Ballinger et al., 2000; McCasland et al. 2006) and negatively impact community participation and quality of life (Gutierrez et al., 2007; Chang et al., 2012). While the exact relationship between the physical demands of wheelchair use and the development of shoulder pathology is not yet fully understood, ergonomics studies consistently suggest that there is a link between highly repetitive tasks and the occurrence of upper extremity pain and injury (Frost et al., 2002; Silverstein et al., 2008). Therefore, to prevent further loss of independence and functional mobility, it is imperative to find ways to preserve shoulder function for the MWC user.

In preparing for this Research Topic in Wheeled Mobility Biomechanics, we were particularly interested in receiving contributions about current research that provided insights into the mechanical demands and performance techniques during tasks associated with MWC use in order to gain a greater insight into upper extremity loading consequences, predictors of pain onset and injury, and identifying strategies that can preserve functional mobility for the MWC user.

In organizing the Research Topic issue, we invited a number of experts who study wheeled mobility from different perspectives with the intent of advancing the knowledge regarding the variables that promote or hinder an individual’s capacity to handle the daily manual wheeled mobility demands. This is highlighted in the contribution by Gil-Agudo and colleagues who provided insights into the acute changes to the shoulder’s soft tissues by evaluating the echographic and kinetic changes in the shoulder joint after MWC propulsion under two different workload settings (Gil-Agudo et al.). Zhao and colleagues presented an analysis of the scapular motion in three common tasks performed by individuals who use a MWC to gain insights into potentially detrimental shoulder kinematics experienced during wheelchair use and related activities (Zhao et al.). To provide a comprehensive approach for MWC prescription, training, and long-term care for children who use a MWC, Slavens and colleagues characterized the upper extremity biomechanics of MWC mobility in children and adolescents during propulsion, starting, and stopping (Slavens et al.). They identified the greatest demand occurring during the starting task, with distinct propulsion patterns that were unlike those seen in adults.

Manual wheelchair Propulsion (WCP) technique is one aspect of wheelchair use that is believed to be associated with upper limb overuse injury (Boninger et al., 2002). Two contributions provided excellent insights into the relationship between propulsion technique and upper limb biomechanics (Dysterheft et al.). First, Dysterheft and colleagues studied the changes in adolescents’ WCP biomechanics pre- and post-video and verbal feedback in order to maximize contact angle, while minimizing stroke frequency at the handrim (Paralyzed Veterans of America Consortium for Spinal Cord Medicine, 2005). Second, to gain insights into the relationship between WCP technique and loading consequences, Russell et al. showed how individuals with paraplegia modify WCP biomechanics to accommodate expected increases in reaction forces generated at the pushrim with self-selected increases in WCP speed.

There is growing theoretical and empirical evidence that fluctuations in movement (i.e., motor variability), including asymmetry between each arm during WCP, are related to musculoskeletal pain. In a perspective paper, Sosnoff and colleagues argue that the variability of WCP is impacted by shoulder pain and recommend inclusion of variability metrics can yield insights into shoulder pain development (Sosnoff et al.). Also, drawing from a large sample size, Soltau et al. establish the validity of bilateral symmetry during MWC propulsion in those without significant upper extremity pain or impairment.

For the MWC user, being able to self-transfer is essential for independence and community participation. But independent transfer, particularly car transfer, is complex, physically demanding, and known to provoke shoulder pain (Fliess-Douer et al., 2012). To gain insights into the relationship between movement technique and shoulder loading in activities associated with MWC use, Haubert and colleagues described techniques and factors influencing car transfer and WC loading for individuals with paraplegia driving their own vehicles and using their personal MWC (Haubert et al.). They provide an evidence-based recommendation for safe and effective car transfer technique for maintaining independence and preserving mobility for the MWC user.

We claim that creation and application of evidence-based strategies aimed at preserving shoulder function must be personalized and must address multiple factors related to ergonomics and equipment selection, performance techniques, and load-bearing capability of the individual. These include recommendations for reducing the mechanical loads and muscular demands through ergonomics, wheelchair selection and configuration, and environmental adaptations and personal factors for increasing the capacity to handle the daily mobility demands (Requejo et al., 2008, 2015). By integrating up-to-date knowledge of the musculoskeletal system, individual’s capacity to generate and withstand external demands, preferred multijoint control strategies including propulsion technique, and repetitive load exposure through biomechanical modeling and simulations, feasible interventions can be identified and implemented (Munaretto et al., 2012, 2013; Slowik et al., 2015, 2016a,b).

In practice, we highlight the need for individualization of the wheelchair prescription process such that the characteristics of the wheelchair matched the functional capacity of the individual. Individually configured MWCs and seating systems can change postural alignment that improves comfort by decreasing pain from poor posture and improves the ability and efficiency to self-propel, prolonging mobility and endurance and preventing the development of secondary problems. An appropriate wheelchair and seating system provides a stable base for using upper and lower extremities for all mobility-related daily activities and, most important, propelling a wheelchair to maintain independent functional mobility to maximize quality of life. What is important is that clinicians must identify the wheelchair characteristics that are crucial for each individual and then identify the appropriate wheelchair that results in a fit that is specific and unique to a single MWC user. The ability to prescribe, order, modify, or configure the frame or components, to achieve a final system that meets the medical and functional needs of the individual, remains a key ingredient for preserving wheeled mobility.

## Author Contributions

Drs. PR and JM-G contributed equally to the writing of the contents of this editorial.

## Conflict of Interest Statement

The authors declare that the research was conducted in the absence of any commercial or financial relationships that could be construed as a potential conflict of interest.
